# A prospective analysis of false positive events in a National Colon Cancer Surveillance Program

**DOI:** 10.1186/1472-6963-14-137

**Published:** 2014-03-27

**Authors:** Knut Magne Augestad, Jan Norum, Johnie Rose, Rolv-Ole Lindsetmo

**Affiliations:** 1 Department of Surgery, Division of Colorectal Surgery, University Hospitals Case Medical Center, Cleveland, OH, USA; 2 Norwegian Center of Integrated Care and Telemedicine, University Hospital of North Norway, Tromsø, Norway; 3 Department of Gastrointestinal Surgery, University Hospital of North Norway, Tromsø, Norway; 4 Northern Norway Regional Health Authority Trust, Bodø, Norway; 5 Institute of Clinical Medicine, Faculty of Health Sciences, University of Tromsø – The Artic University of Norway, Tromsø, Norway; 6 Department of Family Medicine and Community Health, Case Western Reserve University School of Medicine, Cleveland, OH, USA; 7 Case Comprehensive Cancer Center, Cleveland, OH, USA

**Keywords:** Colorectal cancer, Surveillance, Follow-up, False positive test, Positive predictive value, Metastases resection, Cost-effectiveness

## Abstract

**Background:**

The survival benefits of colon cancer surveillance programs are well delineated, but less is known about the magnitude of false positive testing. The objective of this study was to estimate the false positive rate and positive predictive value of testing as part of a surveillance program based on national guidelines, and to estimate the degree of testing and resource use needed to identify a curable recurrence.

**Methods:**

Analysis of clinically significant events leading to suspicion of cancer recurrence, false positive events, true cancer recurrences, time to confirmation of diagnosis, and resource use (radiology, blood samples, colonoscopies, consultations) among patients included in a randomised colon cancer surveillance trial.

**Results:**

110 patients surgically treated for colon cancer were followed according to national guidelines for 1884 surveillance months. 1105 tests (503 blood samples, 278 chest x-rays, 209 liver ultrasounds, 115 colonoscopies) and 1186 health care consultations were performed. Of the 48 events leading to suspicion of cancer recurrence, 34 (71%) represented false positives. Thirty-one (65%) were initiated by new symptoms, and 17 (35%) were initiated by test results. Fourteen patients had true cancer recurrence; 7 resections of recurrent disease were performed, 4 of which were successful R0 metastasis Resections. 276 tests and 296 healthcare consultations were needed per R0 resection; the cost per R0 surgery was £ 103207. There was a 29% probability (positive predictive value) of recurrent cancer when a diagnostic work-up was initiated based on surveillance testing or patient complaints.

**Conclusion:**

We observed a high false positive rate and low positive predictive value for significant clinical events suggestive of possible colorectal cancer relapse in the setting of a post-treatment surveillance program based on national guidelines. Providers and their patients should have an appreciation for the modest positive predictive value inherent in colorectal cancer surveillance programs in order to make informed choices, which maximize quality of life during survivorship. Better means of tailoring surveillance programs based on patient risk would likely lead to more effective and cost-effective post-treatment follow-up.

**Trial registration:**

ClinicalTrials.gov identifier NCT00572143. Date of trial registration: 11^th^ of December 2007.

## Background

Colon cancer is the third most common cancer in the western world, and surgery is the only curative treatment. Approximately one-third of those resected will experience recurrent disease with an expected survival of less than two years [1]. Most patients treated with curative intent are included in some form of surveillance program involving periodic evaluations to detect asymptomatic recurrence. Reviews comparing various surveillance programs have suggested that more intensive surveillance strategies tend to increase five-year overall survival by 5-10% [2,3].

However, all preventive programs have associated costs and risks [4-7]. The survival benefits of a surveillance program for colon cancer survivors are well known, but much less is known about the potential negative impacts for patients and their loved ones [3]. False positive test results may negatively impact quality of life (QoL) by leading patients and family members to believe that recurrence has occurred. On the other hand, false negative tests may result in diagnostic delay during which a potentially curable recurrent cancer may become inoperable. In order to more rationally weigh benefits and harms of post-operative surveillance programs, patients and providers need information to help them better understand the implications of positive and negative test results [6]. In addition, it is important to understand the resource use associated with work-up of suspected recurrence and the benefit which can be expected in terms of opportunities for curative treatment of recurrence.

Previously, we have reported the cost-effectiveness and QoL results from a Norwegian randomized trial comparing general practitioner (GP) versus surgeon-organised colon cancer surveillance according to national guidelines [8,9]. In this work, we focus on the outcomes of individuals who underwent a diagnostic work-up in response to a positive surveillance test result or development of interval symptoms that raised concern for recurrence. These outcomes include whether the initial positive test results or symptoms represented a false positive or whether they served as the first sign of a true recurrence, as well as subsequent resource use for both true and false positives. Among patients with true positive tests or symptoms, we examine the extent to which curative resection of recurrent disease was possible.

## Methods

Data from a randomised trial assessing a colon cancer surveillance program based on Norwegian National Guidelines (ClinicalTrials.gov identifier NCT00572143), was linked to follow-up data from the electronic medical records (EMRs) of four Norwegian hospitals [8,9]. Patients who underwent a diagnostic work-up as a result of a positive surveillance test or symptoms suggesting possible recurrence were identified, and subsequent health care resource utilization was quantified. Patients were enrolled in the surveillance program between June 1, 2007 and December 15, 2011.

### Outcomes

Primary outcomes included 1) Positive predictive value and false positive rates for initial positive surveillance test results or symptoms suggestive of possible recurrence, 2) subsequent resource use for patients with both true and false positives, and 3) whether curative resection of recurrent disease was possible. Secondary outcomes included time from initial detection to confirmation of recurrence, number needed to test per curative resection of recurrence, and cost per curative resection of recurrence.

### Ethics

The Regional Committee for Medical Research Ethics (P REK NORD 79/ 2006) and the Norwegian Data Inspectorate approved the research protocol; all patients provided informed written consent.

### Inclusion and exclusion criteria

Inclusion criteria were age less than 75 years with recent curative surgery for Dukes’ stage A, B or C colon cancer. Patients receiving postsurgical adjuvant chemotherapy (some Dukes’ B and all Dukes’ C) were eligible to participate. Exclusion criteria were age greater than 75 years, membership in a healthcare trust not participating in the trial, inability to provide informed consent, and Dukes’ stage D cancer. Patients were followed for up to 2 years (Table 1).

**Table 1:** Norwegian Gastrointestinal Cancer Group (NGICG) 2007 Surveillance Program

**Examination/test**	**Surveillance cycle (postoperative months)**														
	**1**	**3**	**6**	**9**	**12**	**15**	**18**	**21**	**24**	**30**	**36**	**42**	**48**	**54**	**60**
Chest x-ray			X		X		X		X		X		X		X
Liver ultrasonography			X		X		X		X		X		X		X
Colonoscopy					X								X		
CEA measurement	X	X	X	X	X	X	X	X	X	X	X	X	X	X	X
Clinical examination	X	X	X	X	X	X	X	X	X	X	X	X	X	X	X

### Description of the surveillance program

The GP- and surgeon-followed arms underwent the same surveillance regimens based on Norwegian Gastrointestinal Cancer Group 2007 surveillance guidelines (Table 1). The surveillance period included in analyses included nine surveillance cycles (one month through 24 months postoperatively) with regular clinical examinations, CEA measurement, chest x-ray, contrast-enhanced liver ultrasound and colonoscopy according to the intervals described in Table 1.

### Hospitals, primary and secondary care professionals

Three local hospitals and one university hospital trust participated. Approximately 100 patients with colon cancer are surgically treated annually at these four hospitals. Approximately 550 GPs work in the health care trust.

### Serious clinical events

A serious clinical event (SCE) was defined as an episode leading to suspicion of cancer recurrence. An SCE could be triggered by the reported symptoms (at routine consultation or in the intervals between surveillance visits), the clinical findings at surveillance visits or the findings of surveillance tests. The symptoms and clinical findings initiating a diagnostic check-up could include the following: lesion suspicious for colorectal cancer at colonoscopy or rectal examination; increased carcinoembryonic antigen (CEA) values revealed by repeated measurements; blood in the stool detected by the Hemofec (fecal occult blood) test; unexplained abdominal pain; unexplained weight loss of 5 kg during the previous three months; palpable lymphadenopathy; possible metastatic lesions on chest x-ray, liver ultrasound or CT scan; or other signs or symptoms suggestive of cancer recurrence. The time to recurrent cancer diagnosis was defined as the time from an SCE (dated in the GP referral or hospital electronic medical record [EMR]) until the completion of the ensuing diagnostic work-up.

### Data collection

Data on radiology tests, colonoscopies, blood tests, surgical consultations, surgeries, pathology studies, admissions, and hospital discharges were abstracted from the EMRs of all included patients and were used to identify SCEs. In the case of missing information, the surgeons, GPs, or patients were interviewed by telephone. False positive SCEs were considered to have occurred when an SCE triggered a subsequent diagnostic work-up that ruled out recurrence. We identified successful R0 resections of recurrent local or metastatic disease by examining the surgical and postoperative pathology reports.

### Statistics

Proportions were compared using 2x2 contingency tables, and Chi Squared or Fisher’s exact tests with an alpha level of 0.05. Continuous values were compared using t-tests with a two-sided alpha value of 0.05. The results were expressed using the mean differences for continuous outcomes with the corresponding standard deviations (SD), 95% confidence intervals (CI), and associated p-values. P-values were reported to three decimal places, and p-values less than 0.001 were reported as p < 0.001. The surveillance program’s positive predictive value was defined as the proportion of SCEs that were true positives (i.e., true cancer recurrences).

The economic evaluation of cost per R0 resection was performed from a societal perspective. Cost elements were converted into British pounds (BP £) at a rate of BP £ = 9.39 Norwegian krone (NOK) (http://www.norges-bank.no). Unit costs assigned to health care resources have been previously reported [8]. To address the uncertainty aspect of the cost per R0 metastases resections, we performed a many-inputs/one-output sensitivity analyses, with results expressed in a Tornado chart. All analyses were performed using IBM SPSS Statistics v 19.0 (IBM Company SPSS 2010) and Microsoft Excel for Mac 2011.

## Results

A total of 110 patients surgically treated for colon cancer met the inclusion criteria and were enrolled in the randomised trial. Fifty-five were followed by their GPs and 55 were followed in a surgical outpatient department. Overall, 85 patients (75%; GP 41 vs. surgeon 44) were followed for at least 12 months, and 58 patients (52%; GP 29 vs. surgeon 29) were followed for 24 months. The total surveillance time was 1884 person-months; median follow up time was 17 months. Overall, 1105 tests (colonoscopy n = 115, liver ultrasound n = 209, chest x-ray n = 278, carcinoembryonic antigen n = 503) were performed and 1186 consultations occurred (Table 2).

**Table 2:** Demographics of the patients enrolled in a National CRC Surveillance Program

**Variable**	**Surgeon-surveillance n = 55 (%)**	**GP-surveillance n = 55 (%)**	**Total n = 110 (%)**	**p value**
Mean age (SD)	66.7 (7.3)	64.0 (8.7)	65.4 (8.1)	ns
Male	32 (58.2)	33 (60.0)	65 (59.1)	ns
Female	23 (41.8)	22 (40.0)	45 (40.9)	ns
**Tumour location**				
Coecum	13 (23.6)	13 (23.6)	26 (23.6)	ns
Ascendens	9 (16.3)	5 (9.1)	14 (12.7)	ns
Transversum	4 (7.2)	5 (9.1)	9 (8.1)	ns
Decendens	1 (1.8)	4 (1.8)	5 (4.5)	ns
Sigmoid	28 (50.9)	28 (50.9)	56 (50.9)	ns
**Type of surgery**				
Laparoscopic	14 (25.5)	11 (20.0)	25 (22.7)	ns
Open	41 (74.5)	44 (80.0)	85 (77.3)	ns
**Tumour stage**				
Dukes A	12 (21.8)	11 (20.0)	24 (21.8)	ns
Dukes B	25 (45.5)	30 (54.5)	55 (50.0)	ns
Dukes C	18 (32.7)	14 (25.5)	32 (29.0)	ns
Total surveillance months	942	942	1884	ns
Median surveillance (months)	17	17	17	NA
**Surveillance tests n**	513	592	1105	ns
Carcinoembryonic antigen CEA (%)	203 (39)	300 (51)	503	<0.001
Chest X-ray (%)	150 (29)	128 (21)	278	0.003
Liver ultrasound (%)	110 (21)	99 (17)	209	0.03
Colonoscopy (%)	50 (9)	65 (11)	115	ns
Consultations n	508	678	1186	ns

### Serious clinical events and false positive tests

A total of 48 SCEs were identified; 31 (65%) were initiated by emerging symptoms and 17 (35%) were initiated by test findings. Abdominal pain (n = 14, 29%) and blood in the stool (n = 10, 20%) were the most common presenting complaints. There were no significant differences between surgeons or GPs in terms of SCE occurrence or the time to diagnosis (Table 3). Overall, 34 patients (30% of all trial patients) experienced a false positive event. The positive predictive value (i.e., the probability of a surveillance-initiated SCE being confirmed as true colon cancer recurrence) was 29% (surgeons 36% vs. GPs 23%).

**Table 3:** Serious clinical events with suspicion of cancer recurrence

**Serious clinical event characteristics**	**Surgeon-surveillance**	**GP-surveillance**	**Total**	**p value**
Surveillance (months)	942	942	1884	ns
Interval SCE	12	13	25	ns
Routine SCE	11	12	23	ns
Total SCE	22	26	48	ns
**Symptom initiated SCE**				
Abdominal pain (n)	3	11	14	0.05
Blood in stool (n)	6	4	10	ns
Anaemia (n)	0	1	1	ns
Weight loss (n)	1	1	2	ns
Lymphadenopathy (n)	2	0	2	ns
Other findings (n)	1	1	2	ns
Total	13	18	31	ns
True cancer recurrence	4	3	7	ns
**Test initiated SCE**				
Elevated CEA	2	4	6	ns
Radiology	6	3	9	ns
Colonoscopy	1	1	2	ns
Total	9	8	17	ns
True cancer recurrence	4	3	7	ns
False positive tests and symptoms (%)	14 (25%)	20 (36%)	34 (31%)	ns
Program positive predictive value	0.36	0.23	0.29	ns
**Diagnostics for potential recurrent cancer**				
CEA (repeated)	2	4	7	ns
Chest x-ray	4	3	6	ns
CEUS	2	6	6	ns
Colonoscopy	3	12	15	0.05
CT thorax/abdomen/liver	13	8	21	ns
PET	0	2	2	ns
Consultations	14	23	37	ns
Diagnostic work-up days (SD)	45 (45)	35 (28)	39 (35)	ns

PPV: positive predictive value, which is the proportion of positive test/clinical findings that are true positives (i.e., true cancer recurrences). ns: not significant.

### Clinical presentation of colon cancer recurrence

Of the 48 patients with SCEs, 14 (29%) had colon cancer recurrence. Symptoms were the most common initial indication of recurrence (n = 7), followed by radiologically detected lesions (n = 4) and elevated CEA levels (n = 3) (Table 4).

**Table 4:** Clinical presentation of colon cancer recurrence

**Case no**	**Gender**	**Presenting problem**	**Routine vs. interval**	**Diagnostic tests**	**Metastatic site**	**Time to diagnosis (days)**	**Metastasis surgery**	**Time to surgery (days)**
**GP-surveillance**								
1	F	Elevated CEA	Routine	CEUS	Disseminated	27	No	Inoperable
				PET CT				
2	M	Abdominal pain	Interval	CEUS	Liver	21	No	Inoperable
3	M	Elevated CEA	Routine	CEA	Disseminated	71	No	Inoperable
				CT thorax				
				CT abdomen				
4	M	Metastatic lesion detected at CEUS	Routine	CEUS	Liver	4	Yes	38
				CT thorax				
				CT abdomen				
5	F	Abdominal pain, normal CEA, CT and CEUS, disseminated cancer detected at laparotomy	Interval	CEUS	Disseminated	270	Yes	270
				CT thorax				
				CT abdomen				
6	M	Abdominal tenderness	Interval	Anorectoscopy	Local recurrence	2	Yes	30
				CT thorax				
				CT abdomen				
**Surgeon-Surveillance**								
7	M	Metastatic lesion detected chest x-ray	Routine	CT thorax	Lung	45	Yes	62
				CT abdomen				
8	M	Stoma bleeding	Interval	Colonoscopy	Local and lymph node recurrence	10	No	Inoperable
				CT thorax				
				CT abdomen				
9	M	Weight loss	Routine	CT Thorax	Lung	45	No	Inoperable
		Night sweating		CT abdomen				
10	M	Metastatic lesion detected at chest-x ray	Routine	CT Thorax	Lung	4	Yes	42
				CT abdomen				
11	M	Metastatic lesion detected on CEUS	Routine	MR liver	Liver	3	Yes	43
				CT thorax				
				CT abdomen				
12	F	Abdominal pain	Interval	CT abdomen	Disseminated	16	No	Inoperable
				CT thorax				
13	M	Elevated CEA	Routine	CT thorax	Liver	30	No	Inoperable
				CT abdomen	Lung			
				CT liver				
14	F	Occult blood in faeces	Interval	CT thorax	Liver	31	Yes	35
				CT abdomen				
				CEUS				

### Harms and benefits of cancer surveillance

In this surveillance program, 14 recurrences were detected, seven subsequent surgeries were performed, and four R0 resections of metastases were achieved by surveilling 110 patients over a total of 1884 months. This means that 276 tests and 296 healthcare consultations were needed per R0 resection; the cost per R0 resection was £ 103207.

**Figure 1:**
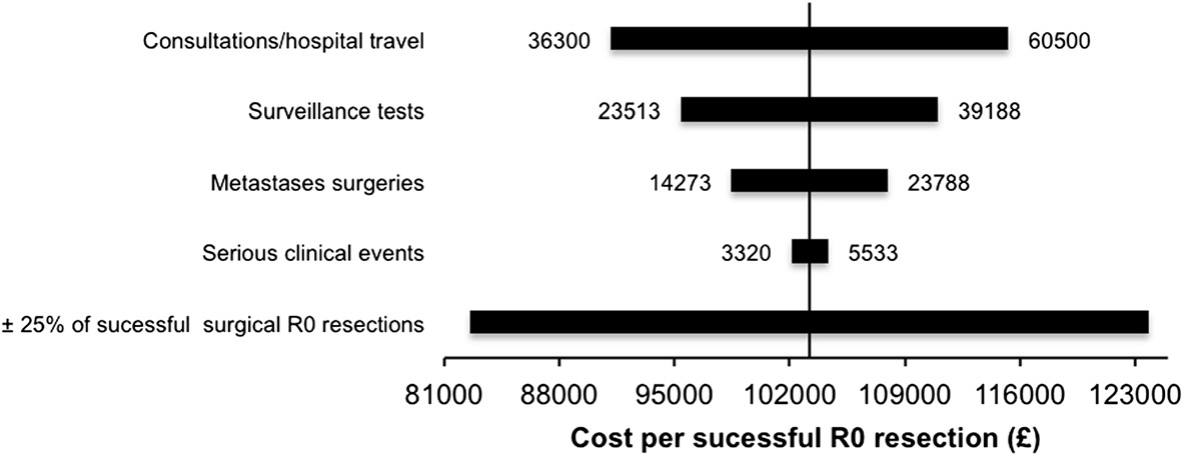
**A sensitivity analysis varying the major cost factors.** The base case per R0 resection was set as origo (£103,000). Variance in the number of successful R0 metastases resections has a major impact on overall cost (range £ 82,566 to £ 123, 847).

A 25% increase in successful R0 metastases surgeries decreased the cost per surgery to £ 82566 (sensitivity analyses Figure 1). Three patients had asymptomatic but incurable recurrences. Mean time from SCE to confirmation of recurrence was 39 days (standard deviation 35 days). The mean quality of life was equal to that of the general UK population (Table 5) [10].

**Table 5:** Fact box of the side-effects and benefits of CRC surveillance

**Variable**	**Surveillance effects**
***Resource use***	
Analysed surveillance months	110 patients for 1884 months
Cost per successful R0 resection (£)	103207
**Screening tests per R0 metastases resection**	
Carcinoembryonic antigen CEA (n)	125
Chest x-ray (n)	70
Liver ultrasound (n)	52
Colonoscopies (n)	30
Total screening tests (n)	276
Total health care consultations (n)	296
***Benefits***	
Number of cancer recurrences detected (n)	14
Probability of R0 metastasis surgery (%)*	57% (4 of 7 metastasis surgeries)
***Side effects***	
False positive events (n)	34 (31%)
Probability of recurrent cancer when diagnostic check up i.e. surveillance positive predictive value	29%
^*a*^Mean days to SCE diagnosis (SD)	39 (35)
Asymptomatic but incurable metastases recurrences	3 of 14 recurrences (21%)
***Quality of life***	
^a^Mean EQ-5D VAS score (CI)	75.9 (74.5-77.3)
^a^Mean EQ-5D Index score (CI)	0.862 (0.84-0.87)

*R0 surgery is defined as positive histological resection margins. ^a^Reported in a previous publication [8]. Equals that of the general UK population [10].

## Discussion

### Summary of findings

A representative population of patients surgically treated for colon cancer was enrolled in a colon cancer surveillance program based on Norwegian national guidelines, with an expected normal variance of demographic factors and colon cancer severity. The patients were followed for up to two years (i.e., the period during which most cancer recurrences manifest). Thirty percent (34 of 110) of all the surveillance patients experienced false positive tests, and the probability of having a cancer recurrence when a diagnostic check-up was initiated was 29%. Overall, 276 tests were needed to save one patient from recurrent colon cancer, and the total cost per successful surgery for recurrence was £ 103207.

### Comparison of existing literature

The potential negative side effects (physical and psychological) and costs of cancer surveillance strategies have not been well delineated [4-7]. None of the studies included in the previous reviews of colorectal cancer surveillance have provided any specific details of the harms (mortality or morbidity) resulting from investigating or treating recurrences [2,3]. However, a potential harm from any secondary prevention program is well recognised to be over-diagnosis and false positive tests [7,11].

Some researchers have investigated the psychological effects of colorectal cancer surveillance [8,12,13]. None of these studies have found deterioration in the patient QoL with surveillance. Nevertheless, in recent meta-analyses, it was shown that anxiety, rather than depression, was a major problem among long-term cancer survivors. It is unknown, however, what impact a cancer surveillance program itself has on anxiety levels [14].

The challenge of postoperative cancer surveillance is that a vast majority of patients must undergo a large number of tests without any benefit, or even with some potential harm, to identify a few patients with curable recurrence. The high rate of false positive tests (n = 34, 30% of all surveillance patients) in this trial was more than we expected and likely negatively impacts the patient’s and family’s QoL. False positive and true positive tests in colorectal cancer surveillance have been addressed in previous reviews [15]. According to Kievit et al., 370 positive surveillance tests (26 true positives, 7%) and 11 surgeries were required to provide one patient with a long-term survival benefit (five years) [15]. In our survey, 1186 tests were performed during the 1884 person-month surveillance period, which equals 276 tests per successful R0 metastasis surgery. This finding aligned with the results from another Norwegian survey, which reported 270 tests per successful R0 resection [16]. The estimated cost of £ 103207 per successful R0 metastases resection is higher than reported by other authors. Kørner et al. reported an estimated cost per R0 resection of £ 15278 (US $ 25289),

Identification of asymptomatic but incurable recurrent disease through surveillance testing raises ethical and quality of life considerations [5,17]. In our study, three patients (21%, Table 4) had asymptomatic but incurable colon cancer recurrence. These figures are somewhat higher than those in a previous study reporting 9% asymptomatic but incurable disease detected in a surveillance program [16].

The imperfect nature of specific surveillance tests themselves (i.e., test sensitivity and specificity) can contribute to the potential harms of surveillance. National surveillance programs are often based on serial CEA measurements, and this biomarker has several pitfalls and shortcomings. A recent study showed that the diagnostic accuracy of serial CEA measurements is low and is impacted by the cut-off value used [18]. Similarly, radiological tests have varying sensitivity and specificity, the latter of which impacts the rate of false positive tests. For example, the rate of false positive tests in CT chest scans has been reported to be as high as 30-50%; as a result, this test is not recommended by some physicians for post-treatment surveillance purposes [19].

Studies of secondary prevention practices around other cancer types have explored potential harmful effects of these programs. In a systematic review addressing screening for lung cancer using thoracic CT scans, most of the detected lung nodules (> 90%) were benign, and invasive nonsurgical procedures in patients with benign lesions were common [20]. Forty-six percent of patients reported psychological distress while awaiting the confirmation of a potential cancer diagnosis [21]. Thus, the potential harms of a preventive program must be carefully weighed against any benefits [20-22]. In the case of colonoscopic colorectal cancer surveillance, the potential harms of the procedure (including significant discomfort, bleeding, and perforation) coupled with the low rate of intraluminal cancer recurrence, have led some to debate whether colonoscopy should be routinely included in surveillance programs [23].

### Strengths and limitations

This study has some strength. It represents the first trial to analyse the potential side effects of colon cancer surveillance, an area of considerable uncertainty. Jeffery and Hider outlined this uncertainty around side effects of colorectal cancer surveillance in their 2007 Cochrane review [3]. This study has limitations. The trial was designed to assess whether general practice surveillance affected patient-specific QoL and cost-effectiveness compared to surgeon-led surveillance [9]. A trial assessing survival would require a larger sample size and a longer surveillance time. We acknowledge that this choice of a follow-up period might have impacted the observed frequency of SCE and thus of recurrence. Thirteen percent (n = 14) of trial patients had colon cancer recurrence; this low recurrence rate was most likely related, at least partly, to the short follow-up duration (median 17 months). Therefore, our calculation of the cost per successful R0 recurrence surgery might represent an overestimate. To address this uncertainty, we performed a sensitivity analysis in which we estimated the cost per R0 resection if a 25% increase in successful resections was observed (Figure 1). However, in our opinion, the analysed time period gives a realistic overview of the magnitude and rate of side effects in a colon cancer surveillance program.

## Conclusion

Information on a range of outcomes should be available to fully assess the net benefit or harm of colon cancer surveillance. Any survival benefit of the surveillance must be balanced against the potential harms inherent to ensure that surveillance programs are acceptable. Providers are best positioned to inform patients and their families of these benefits and potential harms. The nature of postoperative cancer surveillance is that a vast majority of patients (96% in the present study) must undergo a large number of tests without any benefit, or even with some harm to themselves and their family, to identify a few patients with curable recurrence. Patients with asymptomatic but incurable disease (21% of all recurrences in the present study) likely represent the most controversial group and raise ethical and quality of life considerations [16]. The reported cost of £ 103207 per R0 resection of recurrent disease is significantly higher than that reported by others [16].

In conclusion, due to the high rate of false positive tests and low positive predictive value of the surveillance program examined, we feel that a more tailored surveillance approach based on recurrence risk and likely recurrence pattern is needed in future CRC surveillance programs. Such an approach has the potential to reduce costs and the number of false positive tests, while improving positive predictive value. There is nothing in the current evidence base suggesting that such an approach will compromise the potential survival benefit of CRC surveillance. In addition, further research is needed regarding the potential harms of colorectal cancer surveillance, including quality of life impacts due to false positive surveillance tests and to early diagnosis of incurable recurrence. The estimated cost of surveillance is considerable, and whether the identified costs are acceptable when comparing the benefits and harms is a matter of discussion not only among policy and decision makers, but also among providers, patients, and families.

## Competing interests

We declare that we have no competing interests.

## Authors’ contributions

KMA and ROL conceived and designed the research idea and were responsible for the overall administration and direction of the study, the analysis and the data interpretation. KMA performed the statistical analyses. KMA performed the economic analysis with assistance from JN, who contributed to the design, data analysis and interpretation of the findings. KMA wrote the first draft. JR provided substantive revisions and interpretation of results. All of the authors read and approved the final manuscript. KMA had full access to all the data in the study and had final responsibility for the decision to submit the manuscript for publication.

## Pre-publication history

The pre-publication history for this paper can be accessed here:

http://www.biomedcentral.com/1472-6963/14/137/prepub

## References

[B1] Larsen I Cancer registry of Norway Cancer Norway 2011 14 1 90 Available from: http://www.kreftregisteret.no/no/Generelt/Publikasjoner/Cancer-in-Norway/Cancer-in-Norway-2011/

[B2] Tjandra J Chan MKY Follow-up after curative resection of colorectal cancer: a meta-analysis Dis Colon Rectum 2007 14 1783 1799 10.1007/s10350-007-9030-5 17874269

[B3] Jeffery M Hickey BE Hider PN Follow-up strategies for patients treated for non-metastatic colorectal cancer Cochrane Database Syst Rev 2007 14 1 25 10.1002/14651858.CD002200.pub2 17253476

[B4] Marshall KG Prevention. How much harm? How much benefit? 1. Influence of reporting methods on perception of benefits Can Med Assoc J 1996 14 1493 8624999 PMC1487820

[B5] Marshall KG Prevention. How much harm? How much benefit? 2. Ten potential pitfalls in determining the clinical significance of benefits Can Med Assoc J 1996 14 1837 8653643 PMC1487736

[B6] Marshall KG Prevention. How much harm? How much benefit? 3. Physical, psychological and social harm Can Med Assoc J 1996 14 169 8800074 PMC1487962

[B7] Independent UK Panel on Breast Cancer Screening The benefits and harms of breast cancer screening: an independent review Lancet 2012 14 1778 1786 23117178 10.1016/S0140-6736(12)61611-0

[B8] Augestad KM Norum J Dehof S Aspevik R Ringberg U Nestvold T Vonen B Skrøvseth SO Lindsetmo R-O Cost-effectiveness and quality of life in surgeon versus general practitioner-organised colon cancer surveillance: a randomised controlled trial BMJ Open 2013 14 4 doi:10.1136/bmjopen-2012-002391 10.1136/bmjopen-2012-002391 PMC3641467 23564936

[B9] Augestad KM Vonen B Aspevik R Nestvold T Ringberg U Johnsen R Norum J Lindsetmo R-O Should the surgeon or the general practitioner (GP) follow up patients after surgery for colon cancer? A randomized controlled trial protocol focusing on quality of life, cost-effectiveness and serious clinical events BMC Health Serv Res 2008 14 137 10.1186/1472-6963-8-137 18578856 PMC2474836

[B10] Roset M Sample size cacluations using EQ-5D Qual Life Res 1999 14 1 1110457733

[B11] Njor SH Olsen AH Blichert-Toft M Schwartz W Vejborg I Lynge E Overdiagnosis in screening mammography in Denmark: population based cohort study BMJ 2013 14 f1064 f1064 10.1136/bmj.f1064 23444414 PMC3582341

[B12] Wattchow DA Weller DP Esterman A Pilotto LS McGorm K Hammett Z Platell C Silagy C General practice vs surgical-based follow-up for patients with colon cancer: randomised controlled trial Br J Cancer 2006 14 1116 1121 10.1038/sj.bjc.6603052 16622437 PMC2361245

[B13] Gall CA Weller D Esterman A Pilotto L McGorm K Hammett Z Wattchow D Patient satisfaction and health-related quality of life after treatment for colon cancer Dis Colon Rectum 2007 14 801 809 10.1007/s10350-006-0815-8 17285234

[B14] Mitchell A Ferguson DW Gill J Depression and anxiety in long-term cancer survivors compared with spouses and healthy controls: a systematic review and meta-analysis Lancet Oncol 2013 14 1 12 10.1016/S1470-2045(12)70595-8 23759376

[B15] Kievit J Follow-up of patients with colorectal cancer: numbers needed to test and treat Eur J Cancer 2002 14 986 999 10.1016/S0959-8049(02)00061-8 11978524

[B16] Körner H Søreide K Stokkeland PJ Søreide JA Systematic follow-up after curative surgery for colorectal cancer in Norway: a population-based audit of effectiveness, costs, and compliance J Gastrointest Surg 2005 14 320 328 10.1016/j.gassur.2004.09.023 15749591

[B17] Loprinzi C Hayes DD Smith TT Doc, shouldn’t we be getting some tests? J Clin Oncol 2003 14 108s 111s 10.1200/JCO.2003.01.190 12743213

[B18] Körner H Søreide K Stokkeland PJ Søreide JA Diagnostic accuracy of serum-carcinoembryonic antigen in recurrent colorectal cancer: a receiver operating characteristic curve analysis Ann Surg Oncol 2006 14 417 423 17103264 10.1245/s10434-006-9060-6

[B19] Grossmann I Avenarius JKA Mastboom WJB Klaase JM Preoperative staging with chest ct in patients with colorectal carcinoma: not as a routine procedure Ann Surg Oncol 2010 14 2045 2050 10.1245/s10434-010-0962-y 20151212 PMC2899025

[B20] Mirkin JN Benefits and harms of CT screening for lung cancer: a systematic review JAMA: J Am Med Assoc 2012 14 2418 2429 10.1001/jama.2012.5521 PMC3709596 22610500

[B21] van den Bergh K Essink-Bot M Borsboom G Scholten E van Klaveren R de Koning H Long-term effects of lung cancer computed tomography screening on health-related quality of life: the NELSON trial Eur Respir J 2011 14 154 161 10.1183/09031936.00123410 21148229

[B22] Brewer NT Salz T Systematic review: the long-term effects of false-positive mammograms Ann Intern Med 2007 14 502 510 10.7326/0003-4819-146-7-200704030-00006 17404352

[B23] Søreide K Endoscopic surveillance after curative surgery for sporadic colorectal cancer: patient-tailored, tumor-targeted or biology-driven? Scand J Gastroenterol 2010 14 1255 1261 20553114 10.3109/00365521.2010.496492

